# Slight drought during flowering period can improve Tartary buckwheat yield by regulating carbon and nitrogen metabolism

**DOI:** 10.1038/s41598-024-58180-x

**Published:** 2024-04-29

**Authors:** Peiyun He, Jiangyan Min, Zhuolei Tang, Xue Yang, Kaifeng Huang, Xiaoyan Huang

**Affiliations:** School of Life Science, Guizhou Normal University, Guiyang, 550001 People’s Republic of China

**Keywords:** Tartary buckwheat, Drought, Flowering period, Carbon and nitrogen metabolism, Yield, Physiology, Plant sciences

## Abstract

This study aimed to clarify the effects of drought during flowering period on the carbon and nitrogen metabolism, growth, and yield of Tartary buckwheat. Tartary buckwheat cultivar Jinqiao 2 was treated with well-watered (CK), slight soil-drought stress (LD), moderate soil-drought stress (MD), and severe soil-drought stress (SD), with the soil water potential maintained at − 0.02 to − 0.03, − 0.04 to − 0.05, − 0.05 to − 0.06, and − 0.06 to − 0.07 MPa, respectively. With prolonged growth period and an increase in drought stress, the antioxidant enzyme activities and the contents of substances and activities of enzymes related to carbon and nitrogen metabolism in Tartary buckwheat leaves initially increased and then decreased. Meanwhile, the contents of malondialdehyde and superoxide anion showed a continuous. LD treatment induced the highest antioxidant enzyme activities and the contents of substances and activities of enzymes related to carbon and nitrogen metabolism but the lowest contents of malondialdehyde and superoxide anion in Tartary buckwheat leaves. Compared with CK, LD treatment increased the grain number, 1000-grain weight (MTS), and yield per plant by 6.52%, 17.37%, and 12.35%, respectively. In summary, LD treatment can increase the antioxidant enzyme activities and the contents of substances and activities of enzymes related to carbon and nitrogen metabolism, thus enhancing the adaptability of Tartary buckwheat to drought stress and increasing the yield per plant.

## Introduction

As one of the abiotic stresses affecting crop growth and development, drought severely limits agricultural development^1^. It causes an imbalance in the production and removal of reactive oxygen species in plants. The accumulation of excess reactive oxygen species causes the peroxidation of cell membrane lipids, thereby inhibiting plant growth and development. The first line of defense against drought stress in crops is to accumulate osmoregulatory substances. Soluble sugars, soluble proteins, and proline are the most common osmoregulatory substances. Elevated levels of osmoregulatory substances under drought stress increase cytosol concentration and reduce membrane permeability to maintain crop water balance^2^. Carbon and nitrogen metabolism are two of the most important metabolic processes in plants that directly affect plant growth and development^3^. Sucrose synthase (SS) and sucrose phosphate synthase (SPS) are two key enzymes involved in sucrose metabolism and are closely related to sucrose content^4^; drought stress reduces the activity of SS and SPS, affecting sucrose accumulation and translocation in plants and ultimately influencing crop yield^5^. Glutamate synthase (GOGAT), nitrate reductase (NR), and glutamate dehydrogenase (GDH) are key enzymes in crop nitrogen metabolism that help crops cope with drought stress; their activities fully reflect the level of nitrogen assimilation in the crop^6–8^. Cao et al.^9^ found that the activity of NR was inhibited under drought stress. Xu et al.^10^ reported that drought stress reduces NR and GS activity in soybean leaves, and the GOGAT activity shows a trend of initially increasing and then decreasing. Therefore, drought stress can lead to changes in physiological and biochemical processes related to carbon and nitrogen metabolism in the crop, thus affecting the transport and distribution of carbon and the assimilation of nitrogen, which in turn influence the growth, development, and final yield of the crop.

Tartary buckwheat belongs to *Fagopyrum* Mill. of family Polygonaceae and is popular as an important staple food in China with outstanding nutritional and health benefits^11,12^. Tartary buckwheat has a short growth cycle, is highly resistant to drought and barrenness, and is often grown in arid and semi-arid regions of Sichuan, Guizhou, and Gansu in the alpine mountains^12^. Crops respond differently to drought and have diverse water requirements at various growth stages. Most crops have a water-sensitive period when water deficiency can seriously affect their growth^13^. Hou and He^14^ showed that drought stress affects the growth and development of Tartary buckwheat, especially during the flowering period when it has the greatest impact on the growth of buckwheat. According to Dong^15^, the flowering period is the most water-sensitive period for Tartary buckwheat, and the lack of water during this stage can lead to poor grain filling. Therefore, the flowering period is the most water-sensitive period for Tartary buckwheat, and the degree of drought in this period directly affects the yield. Therefore, we hypothesized that drought during the flowering period may affect the growth and yield formation of Tartary buckwheat by regulating its carbon and nitrogen metabolism. However, studies relevant to this hypothesis are lacking. In the present work, Tartary buckwheat cultivar Jinqiao 2 was used as the test material. Four different drought stress treatments were applied to study the effects of drought during the flowering period on the carbon and nitrogen metabolism-related substances and enzyme activities, agronomic traits, and yields of Tartary buckwheat. This work aimed to reveal the relationship between drought and the carbon and nitrogen metabolism of Tartary buckwheat, analyze the effects of drought during the flowering period on the growth and yield, and provide theoretical basis and technical reference for the high-yield cultivation of Tartary buckwheat.

## Materials and methods

### Plant materials

Tartary buckwheat cultivar Jinqiao 2 was supplied by the Research Center of Buckwheat Industry Technology of Guizhou Normal University, Guiyang, Guizhou Province, China (26° 35′ N, 106° 43′ E) in compliance with relevant institutional, national, and international guidelines and legislation. Permission to collect seeds was obtained. The test soil is a floricultural soil containing humus with base fertility of 43.03 mg kg^−1^ organic matter, 160.12 mg kg^−1^ available phosphorus, 76.30 mg kg^−1^ available potassium, and 59.45 mg kg^−1^ available nitrogen. Soil nutrient contents were determined using a multichannel intelligent soil nutrient meter (OK-V24, China).

### Treatment

The experiment was conducted from August to November 2022 at the outdoor potting trial site of the Research Center of Buckwheat Industry Technology, Guizhou Normal University (26° 35′ 31″, 106° 43′ 9″). A canopy was built over the top to protect against rainfall (the walls and roof of the growth chamber were constructed of a translucent polyurethane material that allowed sunlight to be projected and did not allow for temperature control). The monthly average temperatures, sunshine, and rainfall from August to October were 21.7 °C, 1267 h, and 153 min 2018, respectively, and 19.8 °C, 1123 h, and 116 mm in 2019, respectively. The average temperature and relative humidity in the growth chamber during the growing season were 17.0°C and 50.0%, respectively (measured by a hygrometer, model: TES-1360A).

On August 12, 2022, the seeds of Tartary buckwheat with the same size were selected, disinfected with 1.2% HgCl_2_ for 30 min, washed with distilled water, and air-dried, and sowed. Row spacing and seeding were 30 cm and 5.6 g m^−2^, respectively, with 90–100 plants in each square meter^16^. The plants were potted in plastic flower pots that were 2.4 × 0.8 × 0.3 m. The optimum application rates of nitrogen (urea, containing 46% N), phosphorus (calcium superphosphate, containing 14% P_2_O_5_), and potassium (potassium chloride, containing 60% K_2_O) fertilizers were 135, 70, and 5.0 kg·ha^−1^, respectively^11^. The three fertilizers were mixed together and applied as a base fertilizer once. In accordance with a previous study, four treatments were set up as follows: well-watered (CK, maintaining soil water potential at − 0.02 to − 0.03 MPa), slight soil-drought stress (LD, maintaining soil water potential at − 0.04 to − 0.05 MPa), moderate soil-drought stress (MD, maintaining soil water potential at − 0.05 to − 0.06 MPa), and severe soil-drought stress (SD, maintaining soil water potential at − 0.06 to − 0.07 MPa)^17^. The soil water potential values of − 0.02 to − 0.03 MPa, − 0.04 to 0.05 MPa, − 0.05 to 0.06 MPa, and − 0.06 to 0.07 MPa were calculated using the method of Zhou^18^ and corresponded to the water content of the soil in the field from 0 to 12 cm being 80% to 85%, 60% to 65%, 50% to 55%, and 45% to 50% of the field water holding capacity, respectively. Each treatment was planted in one pot with three replications. After sowing, all pots were irrigated normally and the water control treatment was started at the beginning of flowering (September 27, 2022). A negative pressure soil moisture meter (Hainan Lingyun Technology Co., model: PM-300) whose clay head was buried 10–12 cm deep was placed in each pot to monitor the soil water potential. During the experiment, negative pressure soil moisture meter readings were checked at 18:00 each day, and water was replenished promptly to the set soil water potential values. The water was controlled until the end of the growth period. Harvesting was performed when 75%–80% of the Tartary buckwheat grains in each pot reached maturity (October 28, 2022).

### Sampling

Samples were taken at 5, 10, 15, 20, and 25 days from the beginning of flowering. Ten Tartary buckwheat plants with similar growth were randomly selected for agronomic traits from the pots of each treatment. The leaves on the fourth node on the main stem (from top to bottom) of each treatment were collected. After being frozen in liquid nitrogen for 30 s, the leaves were stored in a refrigerator at -80°C.

### Determination

#### Agronomic characters and yield

The plant height, stem thickness, and number of main stem nodes of five Tartary buckwheat plants were measured following the method of Zhou et al.^19^. Grains were harvested from each plot when at least 70–80% of the grains matured. After the grains naturally dried, the grain number per plant, 1000-grain weight (MTS), and grain yield per plant were determined, and the average of five replicates was calculated^16^.

#### Antioxidant enzyme activity and MDA content

Superoxide dismutase (SOD) activity was determined by nitrogen blue tetrazole (NBT) method. 0.5 g of the sample was accurately weighted to prepare SOD crude extract. Phosphoric acid buffer solution, Met solution, NBT solution, EDTA-Na_2_ solution, riboflavin, SOD enzyme crude solution, and distilled water were successively added into a 5 mL test tube in a certain proportion and placed under light for 20 min. SOD activity was the calculated by colorimetry at 560 nm^20,21^. Analysis was done in three replicates.

Peroxidase (POD) activity was determined by guaiacol method. 0.5 g of the sample was weighed and extracted with phosphoric acid buffer. Phosphoric acid buffer, H_2_O_2_, and guaiacol were added and colorimetrized at 470 nm. The enzyme activity unit was 0.01 change of A470 per minute^20,21^. Analysis was done in three replicates.

Malondialdehyde (MDA) content was measured by thiobarbituric acid method. 0.2 g of Tartary buckwheat leaves were weighed, ground evenly with 2 mL of 10% trichloroacetic acid, and centrifuged at 12,000 rpm for 20 min at 4 °C. Afterward, 1 mL of the test solution and 1 mL of reaction solution were added. The mixture was shaken, mixed, and kept at 95 °C for 15 min. The optical density (OD) value at 450, 532, and 600 nm was measured after cooling^20,21^. Analysis was done in three replicates.

Superoxide anion radical (O_2_^−^) content was measured by p-aminobenzenesulfonic acid method. 0.1 g of Tartary buckwheat leaves were weighed, ground evenly with 1.6 mL of 50 mM phosphate buffer (pH7.8) and centrifuged at 1000 rpm for 15 min at 4 °C. Afterward, 0.5 mL of the solution was mixed with 0.5 mL of 50 mM phosphate buffer and 0.1 mL of 10 mM hydroxylamine hydrochloride, followed by incubation at 25 °C for 1 h. The mixture was then added with 1 mL of sulfonamide (58 mM) and 1 mL of α-naphthylamine (7 mM) and incubated at 25 °C for 20 min. After the addition of 3 mL of chloroform, mixing, and standing, the pink supernatant was extracted to determine the light absorption value at 530 nm^21^. Analysis was done in three replicates.

#### Contents of substances and activities of enzymes related to carbon and nitrogen metabolism

Soluble sugar content was measured by anthrone colorimetry. 0.2 g of Tartary buckwheat leaves were weighed, ground evenly with 4 mL of 80% ethanol, and kept in a water bath at 80 °C for 30 min. After centrifugation at 5000 rpm for 10 min, the supernatant was transferred and the residue was added with 2 mL of 80% ethanol for repeated extraction. All of the supernatants were mixed. The activated carbon was added, decolored in a water bath for 30 min, diluted to 10 mL, and filtered to obtain soluble sugar and sucrose solution. Volume 0.5 mL of the solution was mixed with an equal volume of distilled water, followed by the addition of 5 mL of anthrone sulfate solution. The mixture was shaken quickly and boiled for 10 min, and the light absorption value at 620 nm was measured after cooling^22^. Analysis was done in three replicates.

Starch content was measured by anthrone colorimetry. The abovementioned residues were dried at 80 °C, and 2 mL of distilled water was added for gelatinization for 15 min. After cooling, 2 mL of 9200 mM precooled perchloric acid was added and extracted for 15 min. The mixture was added with 4 mL of distilled water, shaken well, and centrifuged at 3000 rpm for 10 min. The concentration of perchloric acid in the above process was changed to 4600 mM, and the volume of distilled water to 6 mL. The above steps were repeated, and the second extraction was carried out. 8 mL of distilled water was added for the third extraction. The supernatant after three extractions was diluted to 25 mL, which was the starch solution to be tested. Volume 1 mL of the test solution and 5 mL of anthrone sulfate solution were added. The mixture was shaken, mixed, and kept at 90 °C for 15 min. The OD value at 620 nm was measured after cooling^22^. Analysis was done in three replicates.

Sucrose content was measured by the resorcinol method. 0.4 mL of the above mentioned test solution and 0.2 mL of 2000 mM NaOH were collected and boiled in water for 5 min. After cooling, 2.8 mL of 30% HCl and 0.8 mL of 0.1% resorcinol reagent were added. The mixture was shaken, mixed, and kept at 80 °C for 10 min. The OD value at 480 nm was measured after cooling^22^. Analysis was done in three replicates.

Invertase activity was determined using the method of He^23^. 0.5 g of fresh Tartary buckwheat leaves were ground with precooled distilled water and diluted to 10 mL. After centrifugation at 4000 rpm for 15 min, the supernatant was collected for analysis. After mixing 1 mL of crude enzyme solution, 2.5 mL of pH6.0 phosphate-buffered saline, and 0.5 mL of 10% sucrose solution, the mixture was incubated in a water bath at 37 °C for 0.5 h. Volume 1 mL of the abovementioned mixture was collected, added with 0.75 mL of 3, 5-dinitrosalicylic acid, and boiled for 5 min. After cooling in an ice-water bath, the mixture was diluted to 10 mL with distilled water, and the OD value at 540 nm was determined. Analysis was done in three replicates.

SS and SPS activities were determined in accordance with the method of Chopra et al.^24^. 0.5 g of fresh Tartary buckwheat leaves were ground evenly with 3 mL of Hepes–NaOH extraction buffer and centrifuged at 10,000 rpm for 10 min. The supernatant was taken as the enzyme solution to be tested. The reaction mixture system consisted of 50 μL of Hepes–NaOH buffer (pH7.5), 20 μL of 50 mM MgCl_2_, 20 μL of 100 mM uridine diphosphate glucose, 20 μL of 100 mM fructose, and 50 μL of crude enzyme extract. The control was replaced with the inactivated enzyme solution. The abovementioned mixture was bathed in water at 30 °C for 0.5 h, and 0.2 mL of 2000 mM NaOH solution was added to terminate the reaction. The mixture was bathed in boiling water for 10 min. After cooling, 1.5 mL of 30% HCl and 0.5 mL of 0.1% resorcinol were added, and the mixture was bathed in water at 80 °C for 10 min. The OD value at 480 nm was recorded. Analysis was done in three replicates.

Nitrate nitrogen content was measured using the method of Tang and Luo^22^. 3 mL of distilled water was added to a mortar containing 0.5 g of fresh Tartary buckwheat leaves for grinding. The grinding solution was extracted in a 100 °C water bath for 0.5 h, and the volume was adjusted to 5 mL after filtration. Volume 0.4 mL of salicylic acid–sulfuric acid solution was added to 0.1 mL of the test solution. The mixture was allowed to stand for 20 min and added with 9.5 mL of 0.08 g mL^−1^ NaOH solution. The light absorption value was measured at a wavelength of 410 nm. Analysis was done in three replicates.

Ammonium nitrogen content was measured using the method of Tang and Luo^22^. 0.5 g of fresh Tartary buckwheat leaves were ground into a homogenate with 5 mL of 10% acetic acid solution, diluted with distilled water to 100 mL, and filtered for later use. Afterward, 2 mL of the test solution was added with 3 mL of ninhydrin hydrate and 0.1 mL of 1% ascorbic acid and heated at 100 °C for 15 min. After cooling, the liquid was diluted to 10 mL with anhydrous ethanol, and the OD value was measured at OD580 nm after shaking. Analysis was done in three replicates.

Soluble protein content was determined by the Coomassie brilliant blue method^22^. 0.1 g of fresh Tartary buckwheat leaves were weighed and extracted with phosphoric acid buffer. After being added with the Coomassie Bright Blue solution, the sample was colorimized at 595 nm. The soluble protein content of the sample was calculated according to the standard curve of bovine serum protein. Analysis was done in three replicates.

NR activity was determined in accordance with the method of Tang and Luo^22^. 0.5 g of fresh Tartary buckwheat leaves were ground with 4 mL of extract and centrifuged at 4000 rpm for 15 min. The supernatant contained the test solution. Volume 0.4 mL of enzyme solution was added to 1.2 mL of 100 mM KNO_3_ phosphate buffer and 0.4 mL of NADH solution and then bathed in water at 25 °C for 0.5 h. Afterward, 1 mL of sulfonamide solution was added to terminate the reaction, and 1 mL of naphthyl vinylamine was added to develop the color for 15 min. After centrifugation, the supernatant was collected and the OD value was measured at 540 nm. Analysis was done in three replicates.

GOGAT activity was determined in accordance with the method of Singh and Srivastava^25^. The extraction method of crude enzyme solution was the same as that of GS. The reaction mixture consisted of 1.5 mL of Tris–HCl buffer (pH 7.6), 0.1 mL of 10 mM KCl, 0.5 mL of 20 mM α-ketoglutarate, 0.2 mL of 3 mM NADH, 0.3 mL of enzyme solution, and 0.4 mL of 20 mM l-glutamine. After the reaction started, the change in OD value was recorded every 20 s at 340 nm for 10 consecutive times, and the change in enzyme activity was measured by decreasing the OD value. Analysis was done in three replicates.

Glutamate dehydrogenase (GDH) activity was determined following the method of Loulakakis and Roubelakis-Angelakis^26^. The extraction method of crude enzyme solution was the same as that of GS. The reaction system contained 2.6 mL of crude enzyme solution, 0.1 mL of ddH_2_O, 0.1 mL of 30 mM CaCl_2_, 0.1 mL of 6 mM NADH, and 0.1 mL of crude enzyme extract. The change in OD value was measured at 340 nm. The OD value was recorded every 20 s and continuously measured 10 times. The change in enzyme activity was measured by taking a section with a stable decrease in OD value. Analysis was done in three replicates.

#### Statistical analyses

Data were collated and analyzed using Excel 2016 and DPS 9.50 analysis software. One-way ANOVA was performed, and means were tested by least significant difference at p = 0.05 (LSD_0.05_).

## Results

### Effects of drought during the flowering period on antioxidant enzyme activity and MAD content

The SOD and POD activities in the Tartary buckwheat leaves increased first and then decreased with the advancement of growth period, and the activity at the 15th day from the beginning of flowering was significantly higher than those at the other periods (Table 1). The MDA and O_2_^−^ contents increased continuously with the advance of growth period; their lowest and highest levels were recorded at the 5th and 25th days from the beginning of flowering, respectively. The difference among the different stages reached a significant level. With the aggravation of drought stress, the SOD and POD activities in the leaves increased first and then decreased and were the highest under LD treatment. With the increase in drought stress, the MDA and O_2_^−^ contents decreased first and then increased. Their lowest and highest contents were found under LD and SD treatments, respectively. The difference among the different treatments reached a significant level.

**Table 1:** Effect of drought during the flowering period on the activities of antioxidant enzymes and the contents of malondialdehyde and superoxide anion free radicals in Tartary buckwheat leaves at different growth periods.

Index	Treatment	Period				
		5d	10d	15d	20d	25d
Superoxide dismutase (SOD, U g^−1^)	CK	84.60 ± 1.86b/C	102.60 ± 0.37b/B	108.59 ± 0.65b/A	63.83 ± 0.74b/D	55.05 ± 0.37b/E
	LD	91.89 ± 1.93a/C	107.31 ± 2.80a/B	121.23 ± 0.38a/A	76.04 ± 0.98a/D	63.61 ± 0.65a/E
	MD	79.25 ± 3.71c/C	94.67 ± 0.98c/B	106.24 ± 1.34c/A	57.40 ± 0.74c/D	48.84 ± 0.00c/E
	SD	64.47 ± 0.37d/C	79.25 ± 3.71d/B	95.96 ± 0.74d/A	51.19 ± 0.38d/D	46.70 ± 0.74d/E
Peroxidase (POD, U g^−1^ min^−1^)	CK	360.00 ± 4.44c/D	440.00 ± 4.44c/B	493.33 ± 4.45c/A	377.78 ± 4.45c/C	324.44 ± 4.44c/E
	LD	476.00 ± 13.78a/D	528.89 ± 4.45a/B	600.00 ± 4.44a/A	502.22 ± 4.45a/C	417.78 ± 0.00a/E
	MD	385.78 ± 3.56b/D	466.67b ± 4.45b/B	537.78 ± 4.45b/A	448.89 ± 4.45b/C	336.89 ± 0.89b/E
	SD	324.44 ± 4.45d/C	342.22d ± 4.45d/B	351.11 ± 4.45d/A	329.78 ± 0.89d/C	297.78 ± 4.45d/D
Malondialdehyde (μmol g^−1^)	CK	8.12 ± 0.26c/E	8.65 ± 0.06c/D	11.05 ± 0.01c/C	13.37 ± 0.02c/B	14.37 ± 0.16c/A
	LD	6.98 ± 0.27d/E	7.62 ± 0.16d/D	9.18 ± 0.00d/C	11.82 ± 0.00d/B	13.05 ± 0.01d/A
	MD	8.67 ± 0.00b/E	9.77 ± 0.27b/D	12.10 ± 0.05b/C	15.68 ± 0.04b/B	17.36 ± 0.38b/A
	SD	10.52 ± 0.39a/D	11.22 ± 0.11a/D	14.57 ± 1.05a/C	16.50 ± 0.61a/B	18.46 ± 0.11a/A
Superoxide anion free radicals (nmol g^−1^)	CK	14.55 ± 0.95c/C	14.97 ± 0.18c/C	16.87 ± 0.18c/B	17.39 ± 0.32c/AB	17.81 ± 0.18c/A
	LD	11.59 ± 1.11d/D	13.70 ± 0.18d/C	14.97 ± 0.18d/B	16.13 ± 0.32d/A	16.76 ± 0.00d/A
	MD	16.65 ± 0.18b/C	16.76 ± 0.32b/C	18.24 ± 0.48b/B	18.87 ± 0.18b/B	19.71 ± 0.66b/A
	SD	18.66 ± 0.32a/D	19.50 ± 0.73a/C	20.13 ± 0.36a/BC	20.66 ± 0.18a/B	22.24 ± 0.48a/A

Data are presented as mean ± standard error of the mean. Small letter in the same column means significant difference at *p* < 0.05, Capital letter in the same row means significant difference at p < 0.05. The small letter before “/” presented the significant difference among treatments and the small letter after “/” presented the difference among different growth period. CK (well-watered, soil water potential maintained at − 0.02 to − 0.03 MPa); LD (slight soil-drought stress, soil water potential maintained at − 0.04 to − 0.05 MPa); MD (moderate soil-drought stress, soil water potential maintained at − 0.05 to − 0.06 MPa) ;SD (severe soil-drought stress, soil water potential maintained at − 0.06 to − 0.07 MPa). 5d, 10d, 15d, 20d, and 25d mean 5th, 10th, 15th, 20th, and 25th days from the beginning of flowering, respectively.

### Effects of drought during the flowering period on the contents of substances and activities of enzymes related to carbon metabolism

The soluble sugar and starch contents in the leaves increased first and then decreased with the advancement of growth period and were significantly higher than those of the other growth period at 15th and 10th days from the beginning of flowering, respectively (Table 2). The sucrose content decreased continuously with the advance of growth period and was significantly higher at the 5th day from the beginning of flowering than those at the other periods. The activities of invertase, sucrose synthase, and sucrose phosphate synthase increased first and then decreased with the advancement of growth period. The activities of invertase and sucrose synthase were the highest at the 15th day from the beginning of flowering, and that of SPS was the highest at the 20th day from the beginning of flowering. With the aggravation of drought stress, the contents of soluble sugar, sucrose, and starch and the activities of invertase, SS, and SPS increased first and then decreased. All of these parameters were the highest under LD treatment and the lowest under SD treatment, and the difference among the treatments reached a significant level.

**Table 2:** Effects of drought during the flowering period on the contents of substances and activities of enzymes related to carbon metabolism in leaves at different growth period.

Index	Treatment	Period				
		5d	10d	15d	20d	25d
Soluble sugars (%)	CK	4.47 ± 0.02c/C	4.68 ± 0.01c/B	4.76 ± 0.08c/A	3.40 ± 0.02c/D	3.22 ± 0.04c/E
	LD	5.31 ± 0.00a/C	5.69 ± 0.08a/B	5.85 ± 0.02a/A	4.16 ± 0.07a/D	3.91 ± 0.06a/E
	MD	4.70 ± 0.01b/C	4.82 ± 0.06b/B	5.07 ± 0.05b/A	3.60 ± 0.01b/D	3.42 ± 0.03b/E
	SD	4.16 ± 0.01d/A	4.33 ± 0.09d/A	4.39 ± 0.23d/A	3.31 ± 0.03d/B	2.88 ± 0.16d/E
Sucrose (mg g^−1^)	CK	1.76 ± 0.03c/A	1.50 ± 0.00c/B	1.32 ± 0.01c/C	1.17 ± 0.00c/D	1.12 ± 0.01c/E
	LD	2.25 ± 0.00a/A	1.92 ± 0.03a/B	1.66 ± 0.00a/C	1.40 ± 0.01a/D	1.36 ± 0.00a/E
	MD	1.95 ± 0.01b/A	1.77 ± 0.01b/B	1.40 ± 0.01b/C	1.28 ± 0.00b/D	1.22 ± 0.00b/E
	SD	1.55 ± 0.01d/A	1.38 ± 0.03d/B	1.21 ± 0.01d/C	1.07 ± 0.01d/D	1.00 ± 0.01d/E
Starch (%)	CK	0.21 ± 0.00b/B	0.23 ± 0.00b/A	0.21 ± 0.00b/B	0.19 ± 0.00b/C	0.18 ± 0.00b/D
	LD	0.22 ± 0.00a/C	0.25 ± 0.00a/A	0.23 ± 0.00a/B	0.22 ± 0.00a/C	0.20 ± 0.00a/D
	MD	0.21 ± 0.00b/B	0.23 ± 0.00b/A	0.21 ± 0.00b/B	0.19 ± 0.00b/C	0.18 ± 0.00b/D
	SD	0.18 ± 0.00c/B	0.19 ± 0.00c/A	0.18 ± 0.00c/B	0.17 ± 0.00c/C	0.14 ± 0.00c/D
Invertase (mg g^−1^ h^−1^)	CK	2.76 ± 0.00b/C	2.88 ± 0.02b/B	3.07 ± 0.00b/A	2.66 ± 0.00b/D	2.58 ± 0.01b/E
	LD	2.86 ± 0.01a/C	3.33 ± 0.02a/B	3.46 ± 0.05a/A	2.88 ± 0.00a/C	2.79 ± 0.00a/D
	MD	2.52 ± 0.00c/D	2.79 ± 0.00c/B	2.91 ± 0.00c/A	2.55 ± 0.02c/C	2.46 ± 0.02c/E
	SD	2.42 ± 0.00d/C	2.56 ± 0.03d/B	2.75 ± 0.02d/A	2.45 ± 0.00d/C	2.30 ± 0.01d/D
Sucrose synthase (SS, mg g^−1^ h^−1^)	CK	30.12 ± 0.00b/D	33.81 ± 0.02b/C	36.83 ± 0.37b/A	34.47 ± 0.44b/B	33.61 ± 0.21b/C
	LD	34.10 ± 0.20a/E	38.07 ± 0.21a/B	41.06 ± 0.13a/A	37.32 ± 0.15a/C	34.85 ± 0.21a/D
	MD	28.69 ± 0.02c/D	30.97 ± 0.00c/C	34.17 ± 0.59c/A	32.74 ± 0.02c/B	30.65 ± 0.21c/C
	SD	26.46 ± 0.02d/E	28.55 ± 0.21d/C	30.57 ± 0.09d/A	29.41 ± 0.00d/B	27.68 ± 0.21d/D
Sucrose phosphate synthase (SPS, mg g^−1^ h^−1^)	CK	35.35 ± 0.37b/C	37.26 ± 0.08b/B	41.08 ± 0.59b/A	42.27 ± 1.97b/A	36.03 ± 0.28b/BC
	LD	37.51 ± 0.02a/E	41.28 ± 0.00a/D	46.13 ± 0.23a/B	50.93 ± 0.23a/A	44.11 ± 0.24a/C
	MD	35.59 ± 0.21b/D	37.41 ± 0.77b/C	41.33 ± 0.23b/B	43.47 ± 0.23b/A	36.21 ± 0.43b/D
	SD	31.30 ± 0.06c/E	31.93 ± 0.06c/D	35.60 ± 0.00c/B	37.73 ± 0.23c/A	33.86 ± 0.00c/C

Data are presented as mean ± standard error of the mean. Small letter in the same column means significant difference at *p* < 0.05, Capital letter in the same row means significant difference at *p* < 0.05. The small letter before “/” presented the significant difference among treatments and the small letter after “/” presented the difference among different growth period. CK (well-watered, soil water potential maintained at − 0.02 to − 0.03 MPa); LD (slight soil-drought stress, soil water potential maintained at − 0.04 to − 0.05 MPa); MD (moderate soil-drought stress, soil water potential maintained at − 0.05 to − 0.06 MPa) ;SD (severe soil-drought stress, soil water potential maintained at − 0.06 to − 0.07MPa). 5d, 10d, 15d, 20d, and 25d mean 5th, 10th, 15th, 20th, and 25th days from the beginning of flowering, respectively.

### Effects of drought during the flowering period on the contents of substances and activities of enzymes related to nitrogen metabolism

With the advance of the growth period, the contents of nitrate nitrogen, ammonium nitrogen, soluble protein, and free amino acid and the activities of NR, GOGAT, and GDH in the leaves initially increased and then decreased (Table 3). Except for the nitrate nitrogen content that was at the highest at the 10th day from the beginning of flowering, the contents of ammonium nitrogen, soluble protein, and free amino acid and the activities of NR, GOGAT, and GDH were the highest at the 15th day from the beginning of flowering. These indexes increased first and then decreased with the increase in drought stress and were the highest under LD treatment and the lowest under HD treatment. The difference among the treatments reached a significant level.

**Table 3:** Effect of drought during the flowering period on the contents of substances and activities of enzymes nitrogen metabolism in leaves at different growth period.

Index	Treatment	Period				
		5d	10d	15d	20d	25d
Nitrate nitrogen (mg g^−1^)	CK	33.93 ± 0.03b/B	34.07 ± 0.03b/A	26.63 ± 0.06b/C	20.98 ± 0.06b/D	18.74 ± 0.06b/E
	LD	35.61 ± 0.00a/B	36.04 ± 0.03a/A	32.17 ± 0.06a/C	25.46 ± 0.06a/D	22.22 ± 0.00a/E
	MD	33.67 ± 0.06c/B	33.87 ± 0.03c/A	26.54 ± 0.06b/C	20.93 ± 0.03b/D	18.61 ± 0.00c/E
	SD	19.85 ± 0.03d/C	24.37 ± 0.08d/A	19.98 ± 0.03c/B	15.26 ± 0.03c/D	14.96 ± 0.06d/E
Ammonium nitrogen (mg g^−1^)	CK	14.59 ± 0.00b/D	16.00 ± 0.10b/B	16.61 ± 0.03b/A	15.29 ± 0.63b/C	14.91 ± 0.28b/CD
	LD	15.97 ± 0.02a/BC	16.19 ± 0.06a/B	17.46 ± 0.02a/A	16.04 ± 0.31a/B	15.69 ± 0.26a/C
	MD	14.40 ± 0.00c/B	14.32 ± 0.02c/BC	16.11 ± 0.02c/A	13.76 ± 0.10c/D	14.01 ± 0.42c/C
	SD	12.76 ± 0.00d/C	13.70 ± 0.00d/B	15.89 ± 0.05d/A	12.35 ± 0.26d/D	11.88 ± 0.08d/E
Soluble protein (mg g^−1^)	CK	11.39 ± 0.21b/E	14.41 ± 0.07b/B	16.52 ± 0.00b/A	13.92 ± 0.00b/C	12.91 ± 0.18b/D
	LD	12.40 ± 0.38a/E	15.15 ± 0.10a/C	17.81 ± 0.01a/A	16.13 ± 0.00a/B	14.41 ± 0.12a/D
	MD	10.95 ± 0.00c/E	13.44 ± 0.08c/B	14.23 ± 0.10c/A	13.22 ± 0.15c/C	12.12 ± 0.08c/D
	SD	10.46 ± 0.10d/D	12.81 ± 0.00d/B	12.99 ± 0.00d/A	11.69 ± 0.01d/C	10.40 ± 0.00d/D
Free amino acids (μg g^−1^)	CK	0.82 ± 0.03b/C	0.83 ± 0.01b/C	1.04 ± 0.00b/A	0.90 ± 0.01b/B	0.73 ± 0.01b/D
	LD	0.91 ± 0.01a/D	0.95 ± 0.02a/C	1.09 ± 0.03a/A	1.02 ± 0.01a/B	0.94 ± 0.01a/C
	MD	0.76 ± 0.01c/D	0.78 ± 0.01c/C	0.87 ± 0.00c/A	0.82 ± 0.01c/B	0.69 ± 0.01c/E
	SD	0.68 ± 0.01d/C	0.71 ± 0.02d/B	0.80 ± 0.00d/A	0.71 ± 0.01d/B	0.60 ± 0.01d/D
NitrateReductase (NR, mg g^−1^ h^−1^)	CK	35.78 ± 1.76b/C	37.46 ± 0.36b/B	40.62 ± 0.21b/A	38.41 ± 0.21b/B	37.93 ± 0.10b/B
	LD	37.99 ± 0.18a/E	39.78 ± 0.62a/D	48.39 ± 0.00a/A	45.94 ± 0.10a/B	43.19 ± 0.00a/C
	MD	34.23 ± 0.18b/E	36.02 ± 0.31b/D	40.38 ± 0.10b/A	38.29 ± 0.10b/B	37.87 ± 0.10b/C
	SD	30.05 ± 0.10c/C	32.20 ± 2.30c/B	36.02 ± 0.18c/A	35.48 ± 0.18c/A	33.39 ± 0.10c/B
Glutamate synthase (GOGAT, mg g^−1^ h^−1^)	CK	40.72 ± 1.86b/C	48.23 ± 0.00b/B	54.66 ± 3.22b/A	31.08 ± 1.86b/D	30.97 ± 0.61b/D
	LD	49.30 ± 0.93a/C	60.98 ± 0.09a/B	64.62 ± 2.75a/A	37.99 ± 1.90a/D	35.10 ± 0.76a/E
	MD	40.72 ± 0.93b/C	49.30 ± 2.46b/B	55.19 ± 2.46b/A	31.61 ± 0.93b/D	31.83 ± 1.70b/D
	SD	35.37 ± 1.61c/B	43.40 ± 1.61c/A	45.44 ± 2.42c/A	27.38 ± 1.53c/C	25.34 ± 0.97c/C
Glutamatedehydrogenase (GDH, mg g^−1^ h^−1^)	CK	16.72 ± 0.64b/D	23.36 ± 2.26b/B	29.15 ± 2.26b/A	25.29 ± 1.34b/B	19.72 ± 0.37b/C
	LD	20.36 ± 1.62a/D	27.43 ± 0.98a/C	38.79 ± 0.37a/A	32.15 ± 0.00a/B	27.01 ± 1.70a/C
	MD	16.29 ± 0.98b/D	20.58 ± 2.23b/C	28.29 ± 2.31b/A	25.29 ± 0.74b/B	19.29 ± 1.70b/C
	SD	12.86 ± 2.95c/C	15.00 ± 1.48c/B	18.43 ± 2.07c/A	15.65 ± 1.34c/B	13.72 ± 0.37c/C

Data are presented as mean ± standard error of the mean. Small letter in the same column means significant difference at *p* < 0.05, Capital letter in the same row means significant difference at *p* < 0.05. The small letter before “/” presented the significant difference among treatments and the small letter after “/” presented the difference among different growth period. CK (well-watered, soil water potential maintained at − 0.02 to − 0.03 MPa); LD (slight soil-drought stress, soil water potential maintained at − 0.04 to − 0.05 MPa); MD (moderate soil-drought stress, soil water potential maintained at − 0.05 to − 0.06MPa) ;SD (severe soil-drought stress, soil water potential maintained at − 0.06 to − 0.07 MPa). 5d, 10d, 15d, 20d, and 25d mean 5th, 10th, 15th, 20th, and 25th days from the beginning of flowering, respectively.

### Effects of drought during the flowering period on the agronomic traits of Tartary buckwheat

The plant height, number of nodes, and stem thickness of the main stem initially increased and then decreased with prolonged growth period and were significantly higher at the 15th day from the beginning of flowering than those at the other periods (Table 4). With the aggravation of drought stress, the plant height and main stem node number showed a continuous decline trend and were significantly higher under CK treatment than those under the other three treatments. The stem thickness initially increased and then decreased with the increase in drought stress and reached the maximum under LD treatment and the minimum under SD treatment. The difference among these treatments reached a significant level.

**Table 4:** Effect of drought during the flowering period on agronomic traits of Tartary buckwheat.

Index	Treatment	Period				
		5d	10d	15d	20d	25d
Plant height (cm)	CK	67.33 ± 1.53a/D	87.00 ± 1.00a/B	96.33 ± 0.58a/A	84.67 ± 0.58a/B	80.33 ± 2.52a/C
	LD	63.33 ± 0.58b/D	67.33 ± 1.53b/C	76.33 ± 1.53b/A	72.33 ± 1.53b/B	64.00 ± 1.00b/D
	MD	57.00 ± 1.00c/B	63.00 ± 1.00c/A	64.00 ± 1.00c/A	62.67 ± 1.53c/A	54.00 ± 2.00c/C
	SD	42.67 ± 2.52d/C	56.33 ± 2.08d/A	57.00 ± 1.00d/A	48.00 ± 2.65d/B	46.33 ± 2.52d/BC
Number of main stem nodes	CK	11.00 ± 0.00a/BC	11.67 ± 0.58a/B	12.67 ± 0.58a/A	11.67 ± 0.58a/B	10.67 ± 0.58a/C
	LD	9.67 ± 0.58b/B	10.33 ± 0.58b/AB	11.33 ± 0.58b/A	10.67 ± 0.58b/AB	9.67 ± 0.58b/B
	MD	9.33 ± 0.58b/B	9.67 ± 0.58b/B	10.67 ± 0.58b/A	10.00 ± 0.00b/AB	9.00 ± 0.00b/B
	SD	7.67 ± 0.58c/B	8.33 ± 0.58c/B	9.33 ± 0.58c/A	8.67 ± 0.58c/AB	8.00 ± 0.00c/B
Stem thickness (cm)	CK	0.23 ± 0.001b/D	0.30 ± 0.002b/A	0.30 ± 0.003b/A	0.27 ± 0.001b/B	0.27 ± 0.001b/C
	LD	0.25 ± 0.001a/E	0.31 ± 0.001a/B	0.32 ± 0.001a/A	0.30 ± 0.000a/C	0.29 ± 0.001a/D
	MD	0.21 ± 0.002c/E	0.25 ± 0.001c/D	0.29 ± 0.001c/A	0.26 ± 0.001c/B	0.26 ± 0.001c/C
	SD	0.19 ± 0.002d/E	0.23 ± 0.001d/B	0.28 ± 0.001d/A	0.21 ± 0.001d/C	0.21 ± 0.001d/D

Data are presented as mean ± standard error of the mean. Small letter in the same column means significant difference at *p* < 0.05, Capital letter in the same row means significant difference at *p* < 0.05. The small letter before “/” presented the significant difference among treatments and the small letter after “/” presented the difference among different growth period. CK (well-watered, soil water potential maintained at − 0.02 to − 0.03MPa); LD (slight soil-drought stress, soil water potential maintained at − 0.04 to − 0.05 MPa); MD (moderate soil-drought stress, soil water potential maintained at − 0.05 to − 0.06 MPa) ;SD (severe soil-drought stress, soil water potential maintained at − 0.06 to − 0.07MPa). 5d, 10d, 15d, 20d, and 25d mean 5th, 10th, 15th, 20th, and 25th days from the beginning of flowering, respectively.

### Effects of drought during the flowering period on yield of Tartary buckwheat

With the aggravation of drought stress, the grain number per plant, MTS, and yield per plant increased first and then decreased (Table 5) and were significantly higher under LD treatment than under the other three treatments. Compared with CK treatment, LD treatment increased the grain number per plant, MTS, and yield per plant by 6.52%, 17.37%, and 12.35%, respectively. Compared with CK treatment, SD treatment decreased grain number per plant, MTS, and yield per plant by 51.09%, 19.16%, and 45.68%, respectively.

**Table 5:** Effect of drought during the flowering period on yield of Tartary buckwheat.

Treatment	Grain number per plant	MTS (g)	Grain weight per plant (g)
CK	55.20 ± 0.84b	16.7 ± 2.1b	0.81 ± 0.06b
LD	58.80 ± 1.30a	19.6 ± 0.8a	0.91 ± 0.02a
MD	40.60 ± 3.21c	16.5 ± 2.0b	0.74 ± 0.12b
SD	27.00 ± 1.87d	13.5 ± 1.6c	0.44 ± 0.04c

Data are presented as mean ± standard error of the mean. Small letter in the same column means significant difference at *p* < 0.05. MTS presented 1000-grain weight. CK (well-watered, soil water potential maintained at − 0.02 to − 0.03 MkPa); LD (slight soil-drought stress, soil water potential maintained at − 0.04 to − 0.05 MPa); MD (moderate soil-drought stress, soil water potential maintained at − 0.05 to − 0.06 MPa) ;SD (severe soil-drought stress, soil water potential maintained at − 0.06 to − 0.07 MPa).

## Discussion

Crops can remove excessive free radicals produced by drought stress by increasing the activity of SOD and POD in vivo to alleviate the damage caused by drought stress^27^. He et al.^17^ found that with the aggravation of drought stress, the SOD and POD activities in Tartary buckwheat leaves showed a trend of increasing first and then decreasing, and the MDA content increased sharply. The present study showed that the antioxidant enzyme activity of Tartary buckwheat leaves increased first and then decreased with the aggravation of drought stress and reached the maximum under LD treatment. The MDA and O_2_^−^ contents decreased first and then increased and reached the lowest under LD treatment. These findings are consistent with the results of the above study. These phenomena likely occurred because under LD treatment, Tartary buckwheat could increase the SOD and POD activities to scavenge excess free radicals due to water deficit and mitigate the hazards of drought stress^27^. Meanwhile, SD treatment cause the production of excessive reactive oxygen species in Tartary buckwheat that cannot be eliminated in time, and the MDA content will increases sharply, damaging the membrane cells and leading to serious damage to the growth and development of Tartary buckwheat. These findings indicated that the buckwheat plant adapts to drought by maintaining high enzyme activities and osmotic pressure-regulating substances levels to adapt to adversity and mitigate the damage caused by adversity.

Under stress conditions, plants initiate regulatory mechanisms to adjust sucrose content and promote carbohydrate redistribution to adapt to the stress environment by altering their SS and SPS activities^28^. The present study revealed that compared with those under CK treatment, the soluble sugar, sucrose, and starch contents significantly increased under LD treatment but significantly reduced under SD treatment. This phenomenon occurs because the cellular water loss in various organs of Tartary buckwheat is not serious under LD treatment, and the soluble sugar content in the organs are increased to strengthen osmoregulation; meanwhile, SD treatment leads to severe cellular water loss and prevents normal groth^29^. The present study also found that the activities of sucrose synthase, sucrose phosphate synthase, and invertase were significantly decreased under SD treatment, which was consistent with the findings of Wang^29^. This phenomenon occurs because the ability of synthesis and catabolism of sucrose are reduced under SD treatment, resulting in the sink organ not receiving sufficient photosynthetic assimilates from the source organ and leading to a reduction in the activity of invertase enzymes and sucrose phosphate synthase. Therefore, severe drought stress affects the accumulation and distribution of carbohydrates, which in turn influence the growth and inhibit the final productivity of Tartary buckwheat.

Nitrogen metabolism has an important role in crop resistance to drought stress and is involved in almost all physiological processes in crops^30^. NH^4+^ uptake is generally increased in most plants under drought stress, and high nitrogen uptake can improve plant drought tolerance^31^. Lawlor et al.^32^ found that drought stress increases effective nitrogen uptake and increases the activity of NR. The results of the current experiment showed that with the increase in drought stress, the contents of substances and activities of enzymes related to nitrogen metabolism showed a trend of increasing first and then decreasing. Compared with those under CK treatment, these indexes increased significantly under LD treatment probably because the latter has no significant effect on the absorption of NO_3_^−^ of the roots and could maintain the normal absorption and nitrogen metabolism of the roots. Meanwhile, MD and SD treatments can significantly reduce the content of substances and activity of enzymes related to nitrogen metabolism in Tartary buckwheat leaves. It may be because MD and SD treatment can inhibit NO_3_^−^ uptake by the root system, thus preventing its transport to the leaves in time; as a consequence, the NR activity in the leaves significantly decreases, and NO_3_^−^ reduction is ultimately suppressed^33^. This phenomenon may also be related to the damage caused by severe soil-drought stress to key photosynthetic sites, such as chloroplast cyst membranes^34^.

Most crops respond to drought stress by changing their external morphological structure^18^. This study found that the plant height and main stem nodes were significantly higher under CK treatment. Meanwhile, SD treatment significantly reduced the plant height, main stem nodes, and stem thickness. These results indicated that Tartary buckwheat can adjust its aboveground morphology to adapt to drought stress^15^. Ye et al.^35^ found that drought stress during the water-sensitive growth period of rice would lead to reduced spike number and low MTS, eventually leading to a reduction in yield. The present study showed that with the aggravation of drought stress, the grains number per plant, MTS, and yield per plant showed a trend of increasing first and then decreasing. The highest values were observed under LD treatment, and the lowest under SD treatment. This finding is consistent with the above results^15^. It may be because that LD in the surface soil during the flowering period stimulates the growth of roots and the absorption of water in the deep soil, helping maintain a good aboveground configuration and thereby increasing the yield potential of Tartary buckwheat. As a consequence, the final yield increases. MD and SD may cause serious damage to the roots of Tartary buckwheat, thus reducing the absorption capacity of water and nutrients, and ultimately leading to a decrease in yield.

## Conclusions

Slight soil-drought stress (LD) treatment promoted the antioxidant enzyme activity, which in turn inhibited the production of excessive reactive oxygen species and prevented cell membrane damage due to the lipid peroxidation of cell membranes. LD treatment increased the content of substance and activities of enzyme related to carbon and nitrogen metabolism; maintained the balance and improved the assimilation ability of carbon and nitrogen metabolism; enhanced the adaptability to drought; and increased the yield of Tartary buckwheat.

## Data Availability

The datasets generated and analysed during the current study are not publicly available due this experiment has only partially completed the phase of the experiment and subsequent experiments are still in progress, but are available from the corresponding author on reasonable request.
